# Cognitive behavioral therapy for anxiety and depression symptoms in people of Parkinson’s disease: a systematic review and meta-analysis

**DOI:** 10.3389/fnagi.2025.1440850

**Published:** 2025-09-05

**Authors:** Xing Yu, Qianhao Wu, Yuewen Liu, Peipei Han, Xiaoyu Chen, Qi Guo

**Affiliations:** ^1^Department of Rehabilitation Medicine, Shanghai University of Medicine and Health Sciences Affiliated Zhoupu Hospital, Shanghai, China; ^2^Department of Rehabilitation Medicine, Graduate School of Shanghai University of Traditional Chinese Medicine, Shanghai, China; ^3^Department of Rehabilitation Medicine, Shanghai University of Medicine and Health Sciences, Shanghai, China

**Keywords:** anxiety, cognitive behavioral therapy, depression, meta, Parkinson’s disease (32). 2.2 search strategy PubMed, Embase, cochrane library, web of science (for relevant English literature)

## Abstract

**Objectives:**

We aim to conducted a systematic review and meta-analysis of published RCTs (Randomized Controlled Trials, RCTs) to quantify the effects of CBT (Cognitive behavioral therapy, CBT), including both remote and in-person modalities, on anxiety, depressive symptoms, and QoL (Quality of Life, QoL) in people with PD (Parkinson’s disease, PD).

**Methods:**

The systematic review and meta-analysis followed the Preferred Reporting Items for Systematic Reviews and Meta-Analysis (PRISMA) guidelines. Eight databases were systematically searched for existing RCTs of CBT in people of PD published in English or Chinese. Searches were updated to February 29, 2024. Methodological quality was appraised with the Cochrane Risk of Bias tool. A meta-analysis of comparative effects was performed using the Review Manager v. 5.4 software.

**Results:**

16 RCTs were included in the study. The studies involved a total of 682 participants, the mean age of participants ranged from 43 to 85. Depression scores of people in the CBT intervention group were significantly improved (SMD: −1.01, 95CI [−1.27, −0.74], *P* < 0.001), the overall meta-analysis result showed that the CBT group had significant improvement in anxiety compared to the control group (SMD: −2.00, 95CI [−2.74, −1.26], *P* < 0.001), results did not show a significant improvement in QoL in CBT group (SMD: −0.40, 95CI [−0.84, 0.04], *P* = 0.08).

**Conclusion:**

This systematic review and meta-analysis provide evidence that CBT intervention significantly improved anxiety and depression in People of PD compared to the control group, whether through offline or remote intervention. No improvement effect of CBT intervention on the QoL of People of PD was found. In the future application of telemedicine, interdisciplinary interventions should be explored to improve the motor and non-motor symptoms and QoL of People of PD.

**Systematic review registration:**

https://www.crd.york.ac.uk/prospero/#recordDetails. Identifier: CRD42024526608.

## 1 Introduction

Parkinson’s disease, a common progressive neurodegenerative disorder, characterized by tremor, rigidity, and bradykinesia, can lead to severe disability and contribute to the increasing global public health burden related to motor, non-motor, and cognitive features, which is related to the deposition of aggregated α-synuclein (Ben-Shlomo et al., 2024; Morris et al., 2024). In the past period of time, non-motor symptoms of PD, such as sleep disorders, anxiety, depression, and cognitive impairments, have been considered to play an important role in the potential pathogenesis and clinical diagnosis of the disease, are the result of a series of systematic pathological involvement (Postuma et al., 2015; Schapira et al., 2017). Anxiety and depression are common non-motor symptoms among people of PD, affecting approximately 31 and 40% of them respectively (Charidimou et al., 2011; Broen et al., 2016). In many cases, non-motor symptoms such as anxiety and depression reflect a low level of dopamine in an off state (Pontone et al., 2022), which may be more disabling than motor symptoms and significantly affect the QoL of people of PD. Emerging evidence highlights anxiety and depression as prodromal markers of PD, preceding motor symptoms by up to 20 years (Weintraub and Mamikonyan, 2019). This prodromal association may stem from early α-synuclein pathology in non-dopaminergic systems (e.g., noradrenergic, serotonergic), which disrupt limbic circuits before motor degeneration (Abou Kassm et al., 2021). Depression is highly prevalent in PD across all stages of the disease, including the prodromal stage, and may shorten life expectancy. Depression may precede motor symptoms by 5 years or more, Prospective historical cohort studies using registry and medical records showed that history of depression is about twice more frequent at the time of PD diagnosis compared with age-matched controls (9.2% vs. 4%), so the depression represents one of the earliest and most frequent alerting symptom across age-related neurodegenerative disorders (Prange et al., 2022). Overall, accurate early identification and diagnosis of anxiety and depressive symptoms can contribute to the early risk management of PD.

**FIGURE 1:**
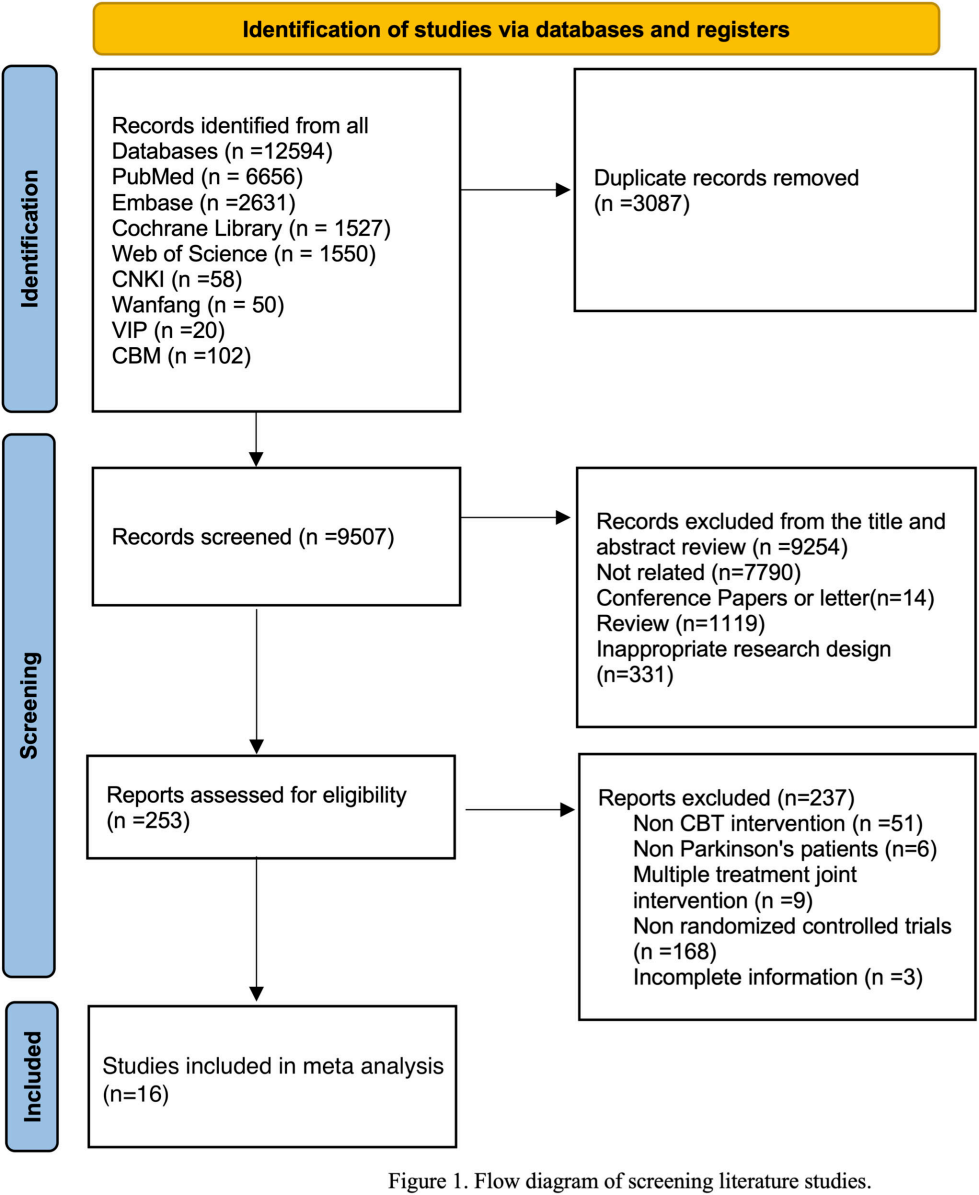
Flow diagram of screening hiterature studies.

A systematic review of 2,399 individuals with PD revealed a point prevalence of 31% for anxiety disorders, with generalized anxiety disorder (GAD): 14.1%, social phobia: 13.8%, and anxiety disorder not otherwise specified (NOS): 13.3% being the most common subtypes. Notably, 31.1% of patients met criteria for two or more concurrent anxiety diagnoses, raising questions about the validity of DSM classifications in PD populations, and probably translating the DSM failure to capture PD-specific anxiety symptomatology (Abou Kassm et al., 2021). Although the DSM fails to fully diagnose PD-specific anxiety, PD-specific anxiety symptoms are more related to fear of progression. Dissanayaka et al. (2016) investigated PD-specific anxiety symptomatology and found frequent insecurity of having PD, worry relating to motor symptoms, social embarrassment and withdrawal due to motor symptoms and “off” periods, and agitation due to motor symptoms and/or complications of PD medications. Dankert et al., (2003) define FoP (fear of progression) as being related to various aspects of the illness itself (Figure 1). Unlike neurotic anxiety disorders (such as generalized anxiety disorder, panic disorder, and agoraphobia), which are characterized by unreal or irrational concerns, FoP represents patients’ fear that the illness will progress with all its biopsychosocial consequences or that it will recur, where patients are confronted with real threats (Herschbach and Dinkel, 2014). The fear arises from personal experiences of a life-threatening or incapacitating illness. Therefore, FoP is an appropriate response to the real threats posed by the diagnosis, treatment, and course of the illness. The level of FoP can range between functional and dysfunctional extremes (Herschbach and Dinkel, 2014). Elevated levels of FoP that become dysfunctional–i.e., those affecting coping mechanisms, treatment adherence, quality of life, or social functioning–require clinical intervention (Herschbach and Dinkel, 2014; Rudolph et al., 2018). Pathological anxiety in PD (linked to noradrenergic/serotonergic dysfunction) must be distinguished from adaptive fear of disease progression. This distinction is critical for targeted interventions. Pathological anxiety in PD often involve symptoms overlapping with autonomic dysfunction, such as thermoregulatory disturbances, hypotension, hyperventilation, and tremors, suggesting unique neurobiological mechanisms (Pontone et al., 2019). Although levodopa drugs may alleviate these symptoms, there are still a considerable number of people of PD who find it difficult to benefit from them (Ray Chaudhuri et al., 2018; Rukavina et al., 2021). Herschbach et al., (2010) found that cancer patients experienced a long-term reduction in FoP following CBT. Furthermore, Sabariego et al. reported that a CBT program targeting FoP demonstrated superior cost-effectiveness compared to other treatment groups (Sabariego et al., 2011).

CBT is a widely used psychotherapy for anxiety, depression, and other mental and psychological disorders. It generally intervenes through the relationship between thinking patterns, feelings, and behaviors. It assumes that psychological problems and emotional distress originate from the way events are interpreted, rather than the events themselves, thus correcting the patient’s negative emotions and cognitions and improving the patient’s unpleasant emotions and inappropriate behaviors (Stallard, 2022). CBT directly targets these features by modifying dysfunctional thinking patterns and maladaptive behaviors, demonstrating efficacy in both anxiety and depression (Carpenter et al., 2018; Cladder-Micus et al., 2018). Previous meta-analyses have explored the efficacy of CBT in alleviating anxiety and depressive symptoms. However, several of these studies were limited by a small number of included trials and insufficient sample sizes (Ghielen et al., 2019; Alnajjar et al., 2024). Furthermore, we note that the number of RCTs continues to grow as CBT protocols evolve and are updated. Additionally, Several studies have investigated the efficacy of non-pharmacological interventions in alleviating anxiety and depressive symptoms (Zhang et al., 2020, 2024; Hong et al., 2021; Wang et al., 2022; Wu et al., 2024), we have noted that meta-analyses on CBT continue to exhibit substantial heterogeneity. Furthermore, it is worth noting that in the past few years, due to the COVID-19 pandemic, clinical visits or offline group sessions have become more difficult in order to reduce patients’ exposure to severe acute respiratory syndrome coronavirus 2 (SARS-CoV-2). A large-scale research report including more than 2,500 people of PD showed that more than 40% of them had aggravated tremor, pain, and mental and psychological symptoms due to the lockdown during the pandemic (Fabbri et al., 2021). The number of people using interdisciplinary telemedicine services has increased exponentially (Podlewska and van Wamelen, 2022). With the advancement of digital services and remote technologies such as brain-computer interfaces, remotely delivered healthcare services are emerging as a prominent trend. Consequently, it is imperative to investigate the effectiveness and safety of remote CBT. Therefore, we conducted a systematic review and meta-analysis of published RCTs to quantify the effects of CBT, including both remote and in-person modalities, on anxiety, depressive symptoms, and QoL in people with PD.

## 2 Methods

### 2.1 Registration and reporting

This study follows the Preferred Reporting Items for Systematic reviews and Meta-analyses (PRISMA 2020) guideline and has registered the research protocol in PROSPERO (registration number: CRD42024526608) (Page et al., 2021).

### 2.2 Search strategy

PubMed, Embase, Cochrane Library, Web of Science (for relevant English literature), China National Knowledge Infrastructure (CNKI), Wanfang Database, China Science and Technology Journal (VIP), and Chinese Biomedical Literature Database (CBM) were systematically searched for existing RCTs of CBT in people of Parkinson’s disease published in English or Chinese. Searches were updated to February 29, 2024. Search terms included “Parkinson Disease” OR “Idiopathic Parkinson’s Disease” OR “Lewy Body Parkinson’s Disease” OR “Parkinson’s Disease, Idiopathic” OR “Parkinson’s Disease, Lewy Body” OR “Parkinson Disease, Idiopathic” OR “Parkinson’s Disease” OR “Idiopathic Parkinson Disease” OR “Lewy Body Parkinson Disease” OR “Primary Parkinsonism” OR “Parkinsonism, Primary” OR “Paralysis Agitans” AND “Cognitive Behavioral Therapy” OR “Behavioral Therapies, Cognitive” OR “Behavioral Therapy, Cognitive” OR “Cognitive Behavioral Therapies” OR “Therapies, Cognitive Behavioral” OR “Therapy, Cognitive Behavioral” OR “Psychotherapy, Cognitive” OR “Therapy, Cognitive” OR “Cognitive Therapies” OR “Therapies, Cognitive” OR “Cognitive Therapy” OR “Cognitive Behavior Therapy” OR “Behavior Therapies, Cognitive” OR “Behavior Therapy, Cognitive” OR “Cognitive Behavior Therapies” OR “Therapies, Cognitive Behavior” OR “Therapy, Cognitive Behavior” OR “Cognitive Psychotherapy” OR “Cognitive Psychotherapies” OR “Psychotherapies, Cognitive” OR “Cognition Therapy” OR “Cognition Therapies” OR “Therapies, Cognition” OR “Therapy, Cognitive Behavior” OR “Behavior Therapies, Cognitive” OR “Cognitive Behavior Therapies” OR “Therapies, Cognitive Behavior” OR “Therapy, Cognition” OR “Behavior Therapy, Cognitive” OR “Cognitive Behavior Therapy”. The detailed search strategy is presented in Supplementary Material.

### 2.3 Inclusion/exclusive criteria

The search for relevant articles was conducted according to the Preferred Reporting Items for Systematic Reviews and Meta-Analyses (PRISMA) flowchart. The inclusion criteria are summarized by the PICOS acronym: (a) participants: clinically diagnosed with PD were included, regardless of anxiety/depression status, disease stage, sex, or age. (b) Intervention: CBT and its modifications, such as iCBT (Internet-based CBT), MBCT (Mindfulness-based Cognitive Therapy) were included, regardless of their modes (face-to-face, Internet, telephone-based or otherwise), form (groups or individuals), the number of sessions, duration, and frequency of each course. (c) Comparison: clinical monitoring only or usual care or none. (d) Outcomes: depression, anxiety, and QoL were measured using standardized instruments, with assessments conducted at least once before and after the intervention. (e) Study design: peer-reviewed RCTs. The exclusion criteria for studies included non-English or non-Chinese studies, participants who were not people of PD, non-RCTs, studies without a control group, studies that employed combined interventions with multiple approaches, and studies with unavailable data.

### 2.4 Data extraction

The data were independently screened and collected by XY and QHW, and any differences were resolved through consultation with YWL. The extracted data from the included studies contained the following information: the name of the first author, the year of publication, characteristics of the subjects, sample size, methodological characteristics, interventions (type, duration, and control details), follow-up time, and other information. All data were collected directly from study tables.

### 2.5 Risk of bias assessment

We assessed the included studies’ quality on the basis of Cochrane Collaboration’s risk of bias tool 2.0 (Sterne et al., 2019). There were three scores for each item (low risk, unclear, and high risk) according to following criteria: (1) risk of bias arising from the randomization process, (2) risk of bias due to deviations from the intended interventions (effect of assignment to intervention), (3) risk of bias due to missing outcome data, (4) risk of bias in measurement of the outcome, (5) risk of bias in selection of the reported result. XY and QHW independently assessed the risk of bias, and any discrepancies were resolved through consultation with YWL.

### 2.6 Statistical analysis

All data and statistical analyses were combined and performed using Review Manager v. 5.4 software (Cochrane Collaboration, Oxford, UK). For each included paper, effect sizes were summarized for each outcome by standard mean differences (SMD), SMD and 95 CI (Confidence Interval) were used to analyze continuous outcomes. We calculated the mean and SD values based on the calculation method mentioned in the Cochrane Handbook version 5.1.0, chapter 16.1.3.2: Imputing Standard deviations for changes from baseline. Based on Cohen’s *d* classification, thresholds of 0.2, 0.5, and 0.8 were applied to categorize SMDs into small, medium, and large effect sizes, respectively. Heterogeneity between studies was tested using chi-square test and Higgins’ I^2^ statistic. A random effects model was used in all meta-analyses, and the fixed effects model as a sensitivity analysis due to the large heterogeneity in study samples, intervention techniques, and assessment instruments throughout the included studies. Sensitivity analysis was performed to identify potential sources of heterogeneity by sequentially omitting one study and investigating the effect of individual studies on the overall pooled estimate, and excluding studies with a high risk of bias. In the presence of significant heterogeneity, we conducted subgroup analyses to identify the sources of heterogeneity. Sensitivity analyses were conducted by excluding each trial on the basis of different subgroups to explore potential sources of heterogeneity. For each of these results with high heterogeneity, separate sensitivity analyses were performed. Potential sources of heterogeneity include characteristics of the subjects, modes of intervention implementation, intervention duration, and so on. Additionally, subgroup analyses were conducted where appropriate to further examine the therapeutic effects of CBT across different age groups, PD with or without diagnosed anxiety or depression, the duration of CBT interventions, and remote CBT versus face-to-face CBT on anxiety and depression. This aims to provide further theoretical evidence for the practical application of CBT. A P-value <0.05 was considered statistically significant.

## 3 Results

### 3.1 Search results and study characteristics

After systematic retrieval in 8 databases, there were 12594 records in total. After removing duplicates, 9507 records were screened by abstracts and titles. After excluding literatures irrelevant to the subject, conference literatures, non-RCTs, etc. 253 literatures were reviewed in full text. Subsequently, 16 RCTs were included in the study (Dobkin et al., 2011, 2020, 2021; Troeung et al., 2014; Moonen et al., 2021), 2 studies employed a health enhancement program (Calleo et al., 2015; Hadinia et al., 2016), 3 studies used standard medical care (Okai et al., 2013; Patel et al., 2017; Kraepelien et al., 2020), 4 studies provided usual care (Xiaohong et al., 2017; Lizhen et al., 2022), and 3 studies utilized a waitlist control (Rodgers et al., 2019; Wuthrich and Rapee, 2019; Piers et al., 2023). The characteristics of the 11 papers included in meta-analyses are presented in Table 1.

**TABLE 1:** Summary of the main details of the reviewed studies.

References	Design	Participant characteristics	Mean age, year	No. I/C	Duration	Intervention details and control conditions	Outcomes
Moonen et al., 2021	Single-blinded RCT	PD with anxiety	I:63.3 ± 7.2 C:63.3 ± 8.4	I:24 C:24	10–12 week (10 weekly standardized individual sessions of 60–75 min, a booster session 6 weeks after the final treatment session)	I: CBT + CM C: CMO	HARS, PAS, LSAS, HDRS, LARS, ZBI, PDQ-8
Hadinia et al., 2016	RCT	PD	I:65 ± 8.7 C:67 ± 11	I:16 C:14	Weekly treatment sessions for the duration of 2 h for 9 weeks	I: CBT C: HEP	PDQ-39, BELA, FKK
Okai et al., 2013	RCT	PD with ICB	I:59.3 ± 8.1 C:57.9 ± 9.5	I:25 C:17	1–12 sessions, Measures were performed at 6 months	I: CBT C: SMC	CGI, NPI, ICBSS, WSAS, GRIMS, GHQ, BDI, BAI
Calleo et al., 2015	RCT	PD with anxiety or depression	62.9 ± 7.3	I:6 C:5	12 weeks (8 skill-based sessions, each lasting 30 – 40 min)	I: CBT C: EUC	SIGH-D, SIGH-A
Troeung et al., 2014	RWCT	PD with anxiety or depression	I: 68 ± 7.72 C: 62 ± 8.34	I:11 C:7	8-week programme consisting of eight 2-h sessions	I: CBT C: CMO	DASS-A, DASS-D, DASS-S, PHQ-39, CCL-D, CCL-A
Kraepelien et al., 2020	RWCT	PD	I: 48–82 C: 43–85	I:38 C:39	10 weeks (one module per week)	I: ICBT C: SMC	WSAS, HADS-A, HADS-D, ISI, PDQ-8, WHODAS-2, BBQ, SSES6
Dobkin et al., 2011	RCT	PD with depression	I: 63.73 ± 9.89 C: 65.44 ± 11.23	I:41 C:39	10 weeks (10 weekly individual sessions (60–75 min))	I: CBT + CM C: CMO	HDRS, BDI, HARS, UPDRS
Dobkin et al., 2020	RCT	PD	I: 65.62 ± 9.76 C: 64.80 ± 9.62	I:37 C:35	3-month (weekly for 10 1-h sessions), monthly during 6-month follow-up	I: T-CBT C: TAU	HAM-D, BDI, HAM-A, Quality of life
Dobkin et al., 2021	RCT	PD with depression	I:67.27 ± 7.79 C:66.42 ± 9.51	I:45 C:45	10 weeks	I: V-CBT C: TAU	HAMD, BDI, HAMA, ATQ, BAS, PCS, Social
Piers et al., 2023	RCT	PD with depression	I:58.5 ± 8.1 C:64.1 ± 5.9	I:6 C:6	12 weekly 50- to 60-min sessions	I: CBT C: Waitlist	MDD Dx CSR, BDI-II
Rodgers et al., 2019	RCT	PD	63.7 ± 8.76	I:18 C:18	8 weeks (six group sessions of 2 h duration each)	I: MBCT C: Waitlist	DASS-A, DASS-D, GAI, GDS-15, PHQ-39
Patel et al., 2017	RCT	PD with insomnia	I: 63.1 ± 6.8 C: 64.7 ± 9.5	I: 14 C: 14	6 weeks	I: CCBT-I C: Standard sleep hygiene education	ESS, FSS, ISI, UPDRS, PDQ, PHQ9, pirs20, EQ5D
Wuthrich and Rapee, 2019	RCT	PD with anxiety or depression	68.82 ± 9.35	I: 5 C: 4	10 weekly manualized sessions 45- min	I: CBT C: Waitlist	GDS, GAI, WHQ-QoL-BRE
Hongmei et al., 2017	RCT	PD	I: 64.64 ± 9.34 C: 65.52 ± 8.65	I:20 C:20	2 weeks (2 sessions/d, 15-30 min/session)	I: CBT C: None	PSQI, HAMD, PDQ39
Lizhen et al., 2022	RCT	PD	I: 66.74 ± 5.39 C: 65.80 ± 5.18	I: 20 C: 17	Unreported	I: CBT C: Usual care	MoCA, MMSE, HAMA, HAMD, PDQ-39
Xiaohong et al., 2017	RCT	PD	I: 47–76 C:46–74	I: 27 C: 25	3-month (1 session/week, 60 min/session)	I: CBT C: Usual care	SAS, SDS, BI

RCT, randomized controlled trial; CBT, cognitive behavior therapy; HEP, health enhancement program; PD, Parkinson disease; I, intervention group; C, control group; HARS, hamilton anxiety rating scale; PAS, Parkinson anxiety scale; LSAS, liebowitz social anxiety scale; HDRS, hamilton depression rating scale; LARS, lille apathy rating scale; ZBI, zarit burden interview; PDQ-8, Parkinson’s disease questionnaire-8; PDQ-39, the Parkinson’s disease questionnaire 39; BELA, burden questionnaire for people of Parkinson’s disease; FKK, questionnaire for disease-related communication; ICB, impulse control behaviors; SMC, standard medical care; BAI, beck anxiety inventory; BDI, beck depression inventory; CGI, clinical global impression; GHQ, general health questionnaire; GRIMS, golombok rust inventory of marital state; ICBSS, impulse control behavior symptom scale; NPI, neuropsychiatric inventory; WSAS, work and social adjustment scale; CMO, clinical monitoring onlyl; EUC, enhanced usual care; RWCT, randomized waitlist-controlled design; DASS-D, DASS-depression; DASS-A, DASS-anxiety; DASS-S, DASS-stress; CCL-D, cognitions checklist-depressive cognitions; CCL-A, cognitions checklist-anxious cognitions; ISI, insomnia severity index; WHODAS-2, world health organization disability assessment schedule 2 - 12-item; BBQ, brunnsviken brief quality of life scale; SSES6, stanford self-efficacy for managing chronic disease; TAU, treatment as usual

### 3.2 Risk of bias

**FIGURE 2:**
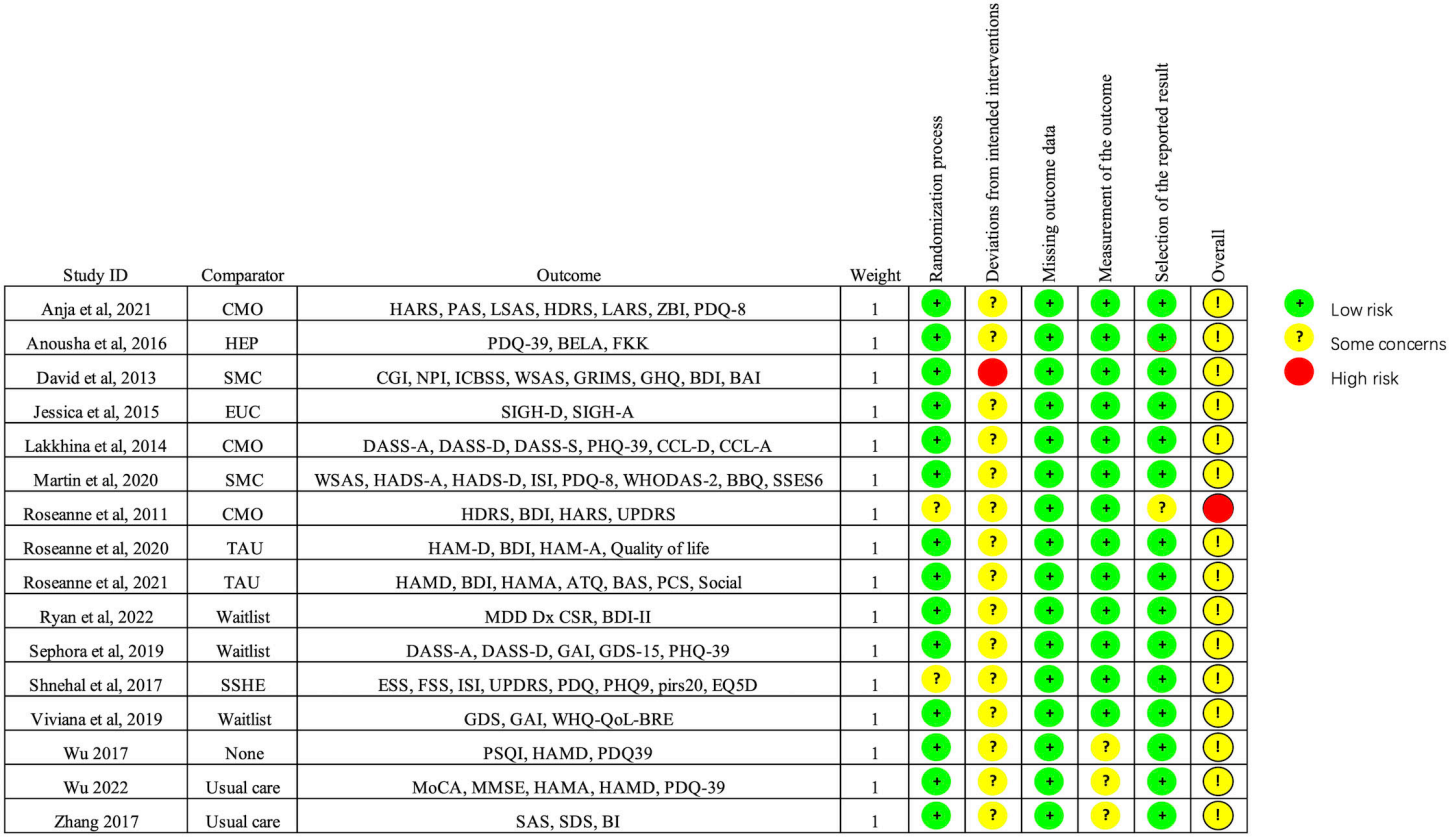
Risk of bias summary.

**FIGURE 3:**
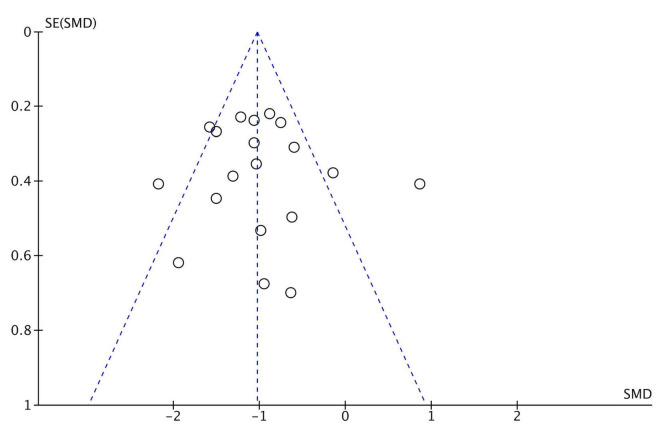
Funnel plot of publication bias.

Figure 2 shows the quality assessment of included studies using the Risk of Bias 2.0 tool as recommended by the Cochrane handbook. (1) Risk of bias arising from the randomization process: 14 studies performed the randomization process well (Okai et al., 2013; Troeung et al., 2014; Calleo et al., 2015; Hadinia et al., 2016; Hongmei et al., 2017; Xiaohong et al., 2017; Rodgers et al., 2019; Wuthrich and Rapee, 2019; Dobkin et al., 2020, 2021), and the other studies were rated as low risk (Okai et al., 2013; Troeung et al., 2014; Calleo et al., 2015; Hadinia et al., 2016; Hongmei et al., 2017; Patel et al., 2017; Xiaohong et al., 2017; Rodgers et al., 2019; Wuthrich and Rapee, 2019; Kraepelien et al., 2020; Moonen et al., 2021; Lizhen et al., 2022; Piers et al., 2023). Overall, the overall risk of bias in 1 study was assessed as high risk (Dobkin et al., 2011). No significant publication bias was found from the funnel plot (Figure 3).

### 3.3 Depression

**FIGURE 4:**
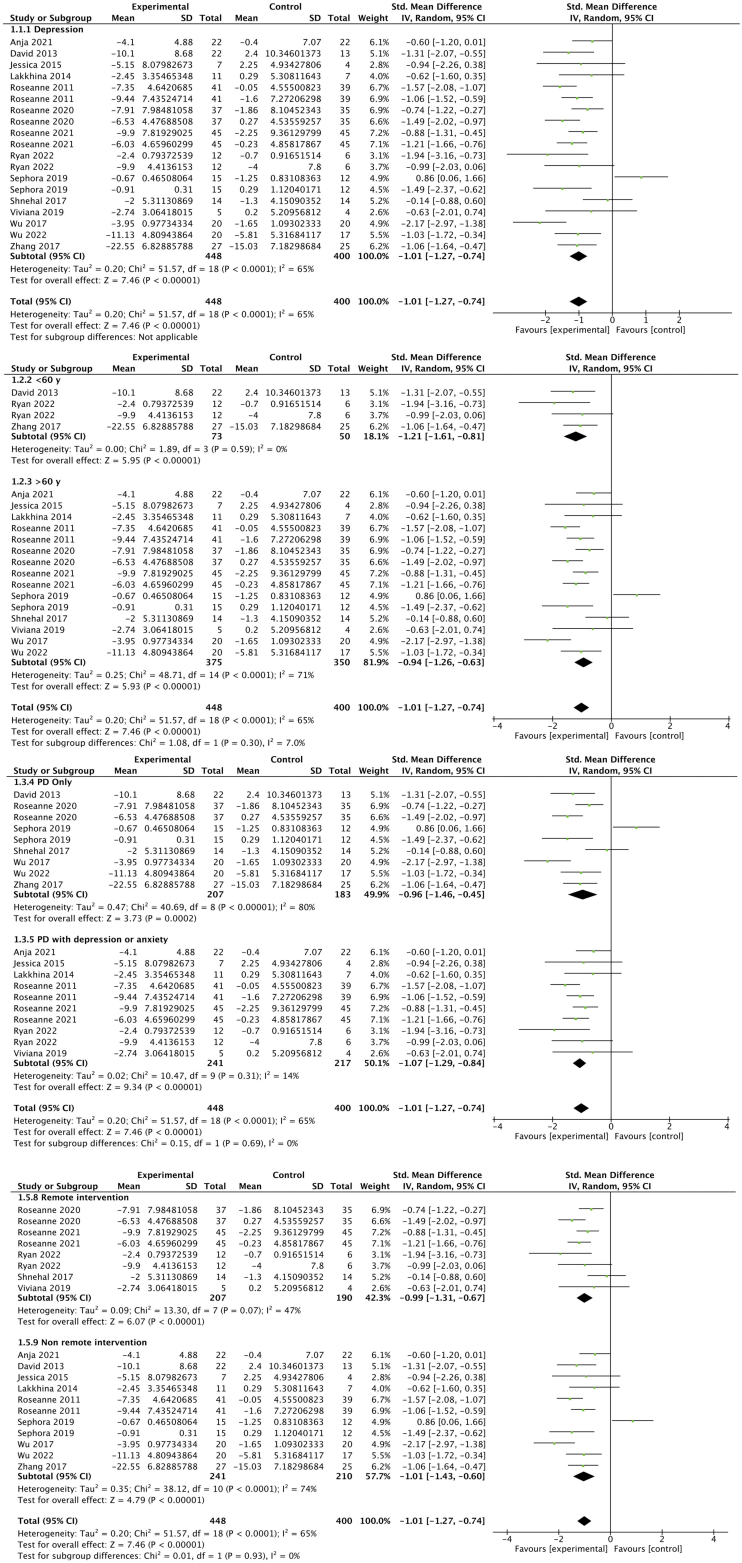
Forest plot of the intervention group versus the control group-depression plot.

A total of 14 studies reported the improvement of depression in people of PD before and after the experiment in the CBT intervention group and the control group (Dobkin et al., 2011, 2020, 2021; Okai et al., 2013; Troeung et al., 2014; Calleo et al., 2015; Hongmei et al., 2017; Patel et al., 2017; Xiaohong et al., 2017; Rodgers et al., 2019; Wuthrich and Rapee, 2019; Moonen et al., 2021; Lizhen et al., 2022; Piers et al., 2023). Since different depression assessment tools were used, SMD was adopted for summary statistics. Random effect model was used to calculate the data when the heterogeneity was high. The comprehensive results showed that the depression scores of people in the CBT intervention group were significantly improved (SMD: −1.01, 95CI [−1.27, −0.74], *P* < 0.001), the effect size falls into the large category (as shown in Figure 4). The I^2^ statistics indicate the presence of substantial heterogeneity, so we conducted subgroup analysis. The subgroup analysis showed that the potential sources of heterogeneity might be the differences in subject age, intervention time, and intervention types. We found that there was no high heterogeneity in the meta-analysis of remote intervention trials, and it showed that the CBT intervention group had a higher improvement in depression compared with the control group (SMD: –0.99, 95CI [–1.31, –0.67], *P* < 0.001). The subgroup analysis results revealed that compared to the subgroup of participants with PD alone (SMD: −0.96, 95% CI [−1.46, −0.45], *P* = 0.0002), the subgroup of participants with PD and anxiety or depression receiving CBT interventions (SMD: −1.07, 95% CI [−1.29, −0.84], *P* < 0.001) showed a larger effect size (SMD: −1.07, 95% CI [−1.29, −0.84], *P* < 0.001), and lower heterogeneity (I^2^ = 14%). The remote CBT intervention subgroup (SMD: −0.99, 95% CI [−1.31, −0.67], *P* < 0.001) and the face-to-face CBT intervention subgroup (SMD: −1.01, 95% CI [−1.43, −0.60], *P* < 0.001) both demonstrated large effect sizes. The remote CBT intervention subgroup exhibited lower heterogeneity (I^2^ = 47) compared to the face-to-face CBT intervention subgroup (I^2^ = 74). Through sensitivity analysis by excluding the literature one by one, there was no difference in the overall results.

### 3.4 Anxiety

**FIGURE 5:**
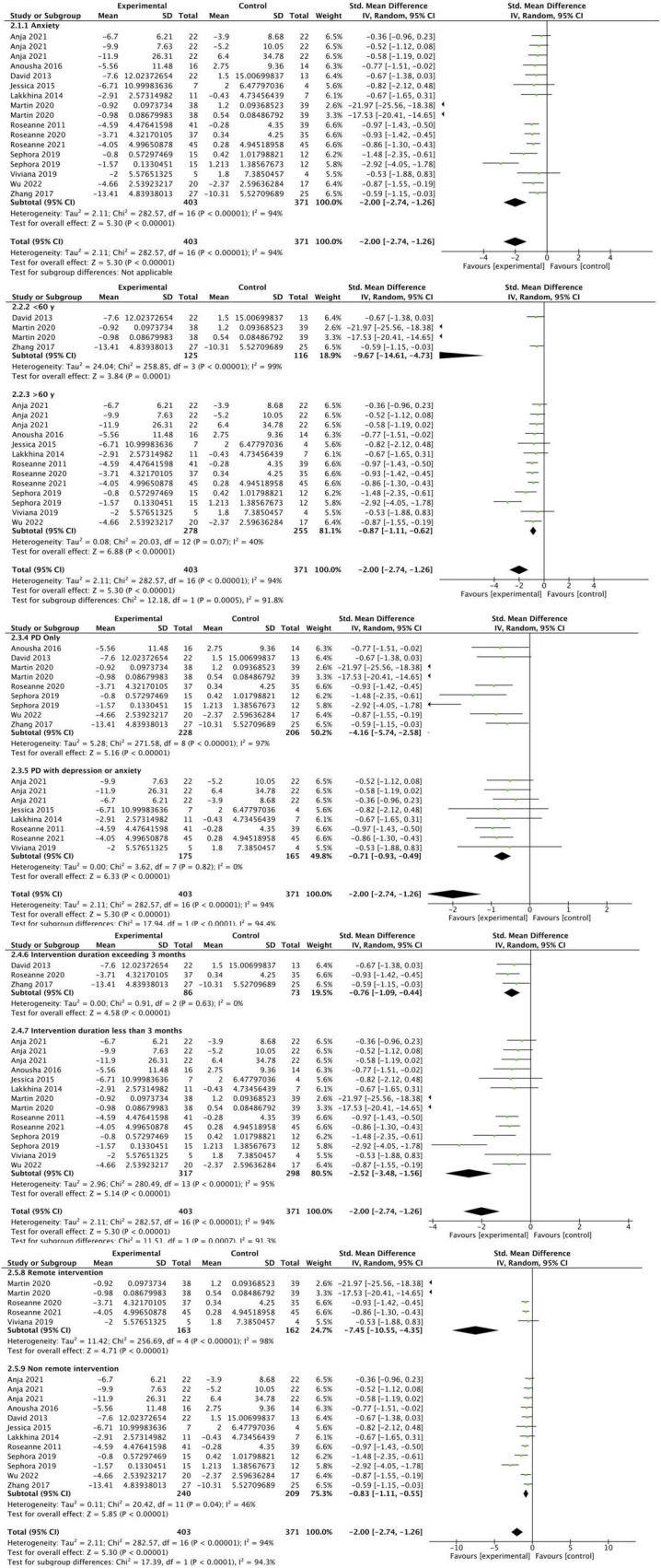
Forest plot of the intervention group versus the control group-anxiety plot.

A total of 13 studies reported anxiety outcomes (Dobkin et al., 2011, 2020, 2021; Okai et al., 2013; Troeung et al., 2014; Calleo et al., 2015; Hadinia et al., 2016; Xiaohong et al., 2017; Rodgers et al., 2019; Wuthrich and Rapee, 2019; Kraepelien et al., 2020; Moonen et al., 2021; Lizhen et al., 2022). The overall meta-analysis result showed that the CBT group had significant improvement in anxiety compared to the control group (SMD: −2.00, 95CI [−2.74, −1.26], *P* < 0.001) the effect size falls into the large category (as shown in Figure 5). Due to the high heterogeneity, we conducted a subgroup analysis, and potential sources of heterogeneity may include differences in intervention types and trial quality. The results of the subgroup analysis showed that the CBT intervention subgroup with PD alone exhibited a larger effect size (SMD: −4.16, 95% CI [−5.74, −2.58], *P* < 0.001) compared to the CBT intervention subgroup with PD comorbid with anxiety or depression (SMD: −0.71, 95% CI [−0.93, −0.49], *P* < 0.001). However, the subgroup with PD comorbid with anxiety or depression demonstrated lower heterogeneity (I^2^ = 0%).Compared to the subgroup with CBT intervention duration less than 3 months (SMD: −2.52, 95% CI [−3.48, −1.56], *P* < 0.001), the subgroup with CBT intervention duration greater than 3 months had a smaller effect size (SMD: −0.76, 95% CI [−1.09, −0.44], *P* < 0.001) and lower heterogeneity (I^2^ = 0%). Both the remote CBT intervention subgroup (SMD: −7.45, 95% CI [−10.55, −4.35], *P* < 0.001) and the face-to-face CBT intervention subgroup (SMD: −0.83, 95% CI [−1.11, −0.55], *P* < 0.001) showed large effect sizes. The remote CBT intervention subgroup exhibited a larger effect size but higher heterogeneity compared to the face-to-face subgroup. We found that after excluding one study (Kraepelien et al., 2020), the heterogeneity was significantly reduced in both the overall results (I^2^ = 33%) and the remote intervention group (I^2^ = 0%), but the results remained unchanged (*P* < 0.001).

### 3.5 Quality of life

**FIGURE 6:**
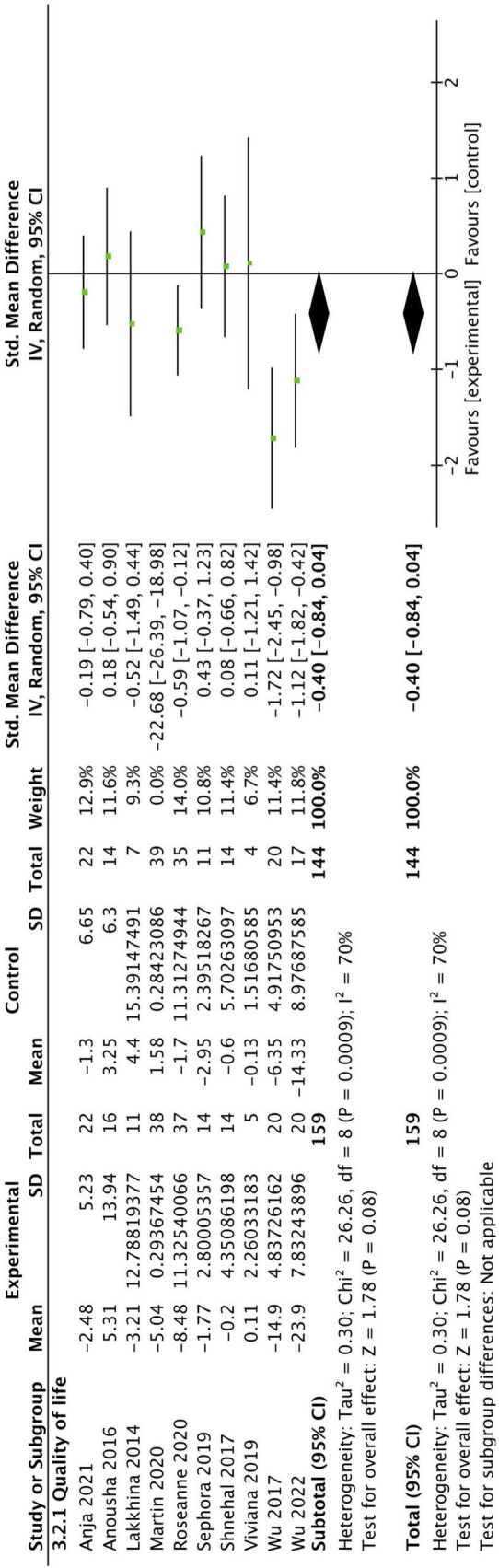
Forest plot of the intervention group versus the control group-quality of life plot.

A total of 10 studies reported QoL outcomes. We excluded one study that had a significant impact on the results. The overall results did not show a significant improvement in QoL in CBT group (SMD: −0.40, 95CI [−0.84, 0.04], *P* = 0.08) (as shown in Figure 6).

## 4 Discussion

We systematically synthesized the existing RCTs on the intervention evaluation of CBT for psychiatric symptoms of People of PD and examined its effectiveness in practice through meta-analysis. Our study found that CBT intervention can effectively improve the depression and anxiety assessment results of people, and the improvement is equally significant in remote CBT intervention. However, no significant improvement was observed in the QoL assessment. Among the 16 studies included, 10 studies were conducted through offline interventions, while 6 studies were conducted remotely via the internet or telephone.

### 4.1 CBT for anxiety and depression in PD

Anxiety is one of the common neurological symptoms of PD. Neuroimaging studies have shown that anxiety in people of PD may be related to the imbalance between two neural circuits (Carey et al., 2021; De Micco et al., 2021). The fear circuit involved in fear processing may be overactivated in people of PD with anxiety, while the limbic-striatal-thalamo-cortical anxiety circuit, a dopaminergic circuit involved in emotional control, may be underactivated (Carey et al., 2023). In addition, white matter (WM) abnormalities are also related to depression (Salehi et al., 2022). Compared with non-depressed people, the brain networks of depressed People of PD involving subcortical, frontal lobe marginal and cortical fibers have changed (Ansari et al., 2019). In people of PD, the development of emotional disorders may precede motor manifestations, and related depression or anxiety issues may not only be secondary to disabilities caused by PD but also share similar pathophysiology (Ishihara and Brayne, 2006), microstructural abnormalities may occur in the fronto-temporal lobes, fronto-limbic, hippocampus, thalamus and its radiations, cerebellum, and WM clusters, and these changes in these regions can significantly affect cognition and consciousness, potentially underlying the cognitive impairments observed in People of PD in previous studies (Salehi et al., 2022).

The results of this meta-analysis show that, CBT has significantly improved depression and anxiety in People of PD. Further subgroup analysis of anxiety improvement in PD patients receiving CBT intervention revealed that the results exhibited lower heterogeneity and greater robustness in subgroups with an average age greater than 60 years, subjects with PD comorbid with depression or anxiety, CBT intervention durations exceeding 3 months, and face-to-face CBT intervention modalities. Subgroup analysis of depression improvement in PD patients receiving CBT intervention revealed that results exhibited lower heterogeneity and greater robustness in subgroups with an average age less than 60 years and those utilizing remote CBT intervention modalities. This suggests that although depression and anxiety may co-occur in PD patients, their underlying neurobiological mechanisms still differ (Wen et al., 2016; Ray and Agarwal, 2020). Common depressive symptoms in PD include sadness, anhedonia, anxiety, and somatic issues (Tanner and Ostrem, 2024). Depression in PD may be associated with greater axial motor symptoms or postural-instability gait disorder subtypes. However, anxiety exhibits a bidirectional relationship with PD motor symptoms–anxiety can exacerbate tremors, and vice versa, and anxiety has been reported more frequently in patients with motor fluctuations and those with younger-onset PD (Goldman, 2025). Therefore, it is necessary to further differentiate and personalize non-pharmacological interventions for the varying psychiatric and psychological symptoms in PD patients.

According to previous studies, “NOS”-type anxiety symptoms include episodic anxiety associated with the wearing-off of dopaminergic medications, as well as anticipatory anxiety characterized by individuals experiencing distress in advance of and often avoiding future events (Dissanayaka et al., 2016). Anxiety in PD frequently manifests with symptoms related to thermoregulation, hypotension, hyperventilation, and trembling, highlighting the need to improve anxiety diagnosis and enhance clinical management (Pontone et al., 2019). Additionally, the classification of anxiety disorders (e.g., PD-specific anxiety diagnoses, panic disorder) is often unclear in reports. Future research should focus on establishing more precise diagnostic criteria for anxiety to facilitate targeted interventions. Most studies employ varying scales to assess depression or anxiety. Moving forward, efforts should be made to standardize assessment tools, particularly by adopting unified psychiatric symptom rating scales specifically developed for PD patients, to reduce research heterogeneity and improve comparability.

### 4.2 CBT for quality of life in PD

We noticed that the meta-analysis results did not show significant improvement in the QoL of people of PD after CBT. In previously published meta-analyses, 2 studies reported significant improvements in QoL for PD patients following CBT (Wu et al., 2024; Zhang et al., 2024). However, these studies only included four RCTs each, with relatively small sample sizes. Notably, they used post-intervention QoL scores rather than pre-post score differences for their meta-analysis, which differs from our calculation method. In contrast, another meta-analysis incorporating 12 RCTs yielded results consistent with ours (Luo et al., 2021), finding no significant improvement in QoL for PD patients after CBT. This study also used pre-post score differences for meta-analysis. Therefore, future research with larger sample sizes and longer intervention durations is still needed to identify effective interventions for improving QoL in PD patients. QoL assessment typically includes several dimensions such as physical activity, social interaction, and emotional well-being. Therefore, improvement in mental health alone is not sufficient to significantly enhance people’ QoL. In the future, multidisciplinary collaboration is still needed to improve motor and non-motor symptoms in people of PD.

### 4.3 Remote CBT and PD

During the COVID-19 pandemic, many offline clinical outpatient visits or other treatment activities were closed down to reduce exposure, and telemedicine became an alternative option. As a result, we have shifted our focus from the hasty and makeshift switch from face-to-face consultations to telemedicine at the onset of the pandemic, to a worldwide surge in the utilization of telemedicine for movement disorders and PD, with a certain level of consensus on the mode of delivery (Hassan et al., 2020). Previous studies have found that although Lewy body pathology is similar across different ethnic groups, the diagnosis and treatment of PD have not received sufficient attention in areas and ethnic groups with poor access to healthcare (Ben-Shlomo et al., 2024). Therefore, due to the disparities in healthcare, providing remote medical guidance can help more people benefit. The implementation and increasing availability of telemedicine have enabled healthcare professionals to provide services to people who would otherwise be unable to access care.

However, while telemedicine can reduce offline contact and distance restrictions, people living in areas without access to internet connection or those with lower socio-economic status may still have limited access to treatment, thus widening the so-called digital divide (Reed et al., 2020), posing new challenges to the methods of remote intervention. A study conducted in rural areas of the United States evaluated the role of remote data collection in the medical treatment of motor and non-motor symptoms of PD, and confirmed the effectiveness of remote assessment through longitudinal evaluation (Virmani et al., 2022). Several previous studies, consistent with the current research, have found that telemedicine interventions significantly improve non-motor symptoms in neurodegenerative diseases (Benge et al., 2020; Maggio et al., 2024). The subgroup analysis of this study revealed that PD patients receiving remote CBT interventions exhibited significant improvements in both anxiety and depressive symptoms, with the results for depression improvement showing lower heterogeneity and greater robustness. The included studies involved remote CBT delivered via computer-based (Patel et al., 2017; Kraepelien et al., 2020; Dobkin et al., 2020, 2021; Piers et al., 2023) and telephone (Wuthrich and Rapee, 2019); however, further RCTs are still needed to confirm the consistency of their effects. The results of this meta-analysis show that the overall QoL of people of PD requires a multidisciplinary approach to intervene in motor and non-motor symptoms, which poses requirements for telemedicine equipment such as sensors, internet access, geolocation data, notifications, and clinical apps (Putzer and Park, 2012).

### 4.4 Strengths and limitations

This systematic review synthesizes findings from previously published RCTs on the efficacy of CBT for anxiety, depression, and quality of life in PD patients. We observed that both remote and face-to-face CBT significantly improved anxiety and depressive symptoms in PD patients. Subgroup analyses further explored the impact of different CBT intervention durations and the efficacy of CBT under varied participant diagnostic profiles. Although this review adhered to meta-analysis guidelines, several limitations should be noted. First, most included RCTs lacked detailed reporting on PD-specific clinical characteristics (e.g., disease duration, staging), precluding further stratified analyses. Second, the absence of standardized anxiety diagnostic classifications limited our ability to draw definitive conclusions about intervention specificity. Third, heterogeneity persisted in the meta-analysis due to variability in intervention protocols and assessment scales, even after subgroup analyses to trace potential sources.

## 5 Conclusion

This study found that CBT intervention significantly improved anxiety and depression in People of PD compared to the control group, whether through offline or remote intervention. No improvement effect of CBT intervention on the QoL of People of PD was found. In the future application of telemedicine, interdisciplinary interventions should be explored to improve the motor and non-motor symptoms and QoL of People of PD. Future research should prioritize larger sample sizes, more rigorous RCT designs, and longer-term follow-up to address these limitations. Additionally, standardized reporting of PD clinical features and anxiety subtypes, along with harmonized assessment tools, is critical to enhance comparability and reduce heterogeneity in future studies.

## Data Availability

The raw data supporting the conclusions of this article will be made available by the authors, without undue reservation.
