# Supplementary material for: Cognitive behavioral therapy for anxiety and depression symptoms in people of Parkinson’s disease: a systematic review and meta-analysis
Source: Front Aging Neurosci. 2025 Sep 5;17:1440850. doi: 10.3389/fnagi.2025.1440850 (PMC12447579; doi:10.3389/fnagi.2025.1440850)
Supplement: Supplementary file 1 [file Table_1.docx]

Supplementary Material:

Pubmed: 6656

(((((((((((((((((((((((((((((("Cognitive Behavioral Therapy"[Mesh]) OR (Behavioral Therapies, Cognitive)) OR (Behavioral Therapy, Cognitive)) OR (Cognitive Behavioral Therapies)) OR (Therapies, Cognitive Behavioral)) OR (Therapy, Cognitive Behavioral)) OR (Psychotherapy, Cognitive)) OR (Therapy, Cognitive)) OR (Cognitive Therapies)) OR (Therapies, Cognitive)) OR (Cognitive Therapy)) OR (Cognitive Behaviour Therapy)) OR (Behaviour Therapies, Cognitive)) OR (Behaviour Therapy, Cognitive)) OR (Cognitive Behaviour Therapies)) OR (Therapies, Cognitive Behaviour)) OR (Therapy, Cognitive Behaviour)) OR (Cognitive Psychotherapy)) OR (Cognitive Psychotherapies)) OR (Psychotherapies, Cognitive)) OR (Cognition Therapy)) OR (Cognition Therapies)) OR (Therapies, Cognition)) OR (Therapy, Cognitive Behavior)) OR (Behavior Therapies, Cognitive)) OR (Cognitive Behavior Therapies)) OR (Therapies, Cognitive Behavior)) OR (Therapy, Cognition)) OR (Behavior Therapy, Cognitive)) OR (Cognitive Behavior Therapy)) AND (((((((((((("Parkinson Disease"[Mesh]) OR (Idiopathic Parkinson's Disease)) OR (Lewy Body Parkinson's Disease)) OR (Parkinson's Disease, Idiopathic)) OR (Parkinson's Disease, Lewy Body)) OR (Parkinson Disease, Idiopathic)) OR (Parkinson's Disease)) OR (Idiopathic Parkinson Disease)) OR (Lewy Body Parkinson Disease)) OR (Primary Parkinsonism)) OR (Parkinsonism, Primary)) OR (Paralysis Agitans))

Web of science:1550

#1 Parkinson Disease (Topic) or Idiopathic Parkinson's Disease (Abstract) or Lewy Body Parkinson's Disease (Abstract) or Parkinson's Disease, Idiopathic (Abstract) or Parkinson's Disease, Lewy Body (Abstract) or Parkinson Disease, Idiopathic (Abstract) or Parkinson's Disease (Abstract) or Idiopathic Parkinson Disease (Abstract) or Lewy Body Parkinson Disease (Abstract) or Primary Parkinsonism (Abstract) or Parkinsonism, Primary (Abstract) or Paralysis Agitans (Abstract)

#2 Cognitive Behavioral Therapy (Topic) or Behavioral Therapies, Cognitive (Abstract) or Behavioral Therapy, Cognitive (Abstract) or Cognitive Behavioral Therapies (Abstract) or Therapies, Cognitive Behavioral (Abstract) or Therapy, Cognitive Behavioral (Abstract) or Psychotherapy, Cognitive (Abstract) or Therapy, Cognitive (Abstract) or Cognitive Therapies (Abstract) or Therapies, Cognitive (Abstract) or Cognitive Therapy (Abstract) or Cognitive Behaviour Therapy (Abstract) or Behaviour Therapies, Cognitive (Abstract) or Behaviour Therapy, Cognitive (Abstract) or Cognitive Behaviour Therapies (Abstract) or Therapies, Cognitive Behaviour (Abstract) or Therapy, Cognitive Behaviour (Abstract) or Cognitive Psychotherapy (Abstract) or Cognitive Psychotherapies (Abstract) or Psychotherapies, Cognitive (Abstract) or Cognition Therapy (Abstract) or Cognition Therapies (Abstract) or Therapies, Cognition (Abstract) or Therapy, Cognitive Behavior (Abstract) or Behavior Therapies, Cognitive (Abstract) or Cognitive Behavior Therapies (Abstract) or Therapies, Cognitive Behavior (Abstract) or Therapy, Cognition (Abstract) or Behavior Therapy, Cognitive (Abstract) or Cognitive Behavior Therapy (Abstract)

#1 AND #2

Cochrane 1527

#1

Parkinson Disease or Idiopathic Parkinson's Disease or Lewy Body Parkinson's Disease or Parkinson's Disease, Idiopathic or Parkinson's Disease, Lewy Body or Parkinson Disease, Idiopathic or Parkinson's Disease or Idiopathic Parkinson Disease or Lewy Body Parkinson Disease or Primary Parkinsonism or Parkinsonism, Primary or Paralysis Agitans

Cognitive Behavioral Therapy or Behavioral Therapies, Cognitive or Behavioral Therapy, Cognitive or Cognitive Behavioral Therapies or Therapies, Cognitive Behavioral or Therapy, Cognitive Behavioral or Psychotherapy, Cognitive or Therapy, Cognitive or Cognitive Therapies or Therapies, Cognitive or Cognitive Therapy or Cognitive Behaviour Therapy or Behaviour Therapies, Cognitive or Behaviour Therapy, Cognitive or Cognitive Behaviour Therapies or Therapies, Cognitive Behaviour or Therapy, Cognitive Behaviour or Cognitive Psychotherapy or Cognitive Psychotherapies or Psychotherapies, Cognitive or Cognition Therapy or Cognition Therapies or Therapies, Cognition or Therapy, Cognitive Behavior or Behavior Therapies, Cognitive or Cognitive Behavior Therapies or Therapies, Cognitive Behavior or Therapy, Cognition or Behavior Therapy, Cognitive or Cognitive Behavior Therapy

#1 and #2

EMBASE 2631

#1

'parkinson disease'/exp OR 'parkinson disease' OR (parkinson AND ('disease'/exp OR disease)) OR 'idiopathic parkinsons disease':ti,ab,kw OR 'lewy body parkinsons disease':ti,ab,kw OR 'parkinsons disease, idiopathic':ti,ab,kw OR 'parkinsons disease, lewy body':ti,ab,kw OR 'parkinson disease, idiopathic':ti,ab,kw OR 'parkinsons disease':ti,ab,kw OR 'idiopathic parkinson disease':ti,ab,kw OR 'lewy body parkinson disease':ti,ab,kw OR 'primary parkinsonism':ti,ab,kw OR 'parkinsonism, primary':ti,ab,kw OR 'paralysis agitans':ti,ab,kw

#2

'cognitive behavioral therapy'/exp OR 'cognitive behavioral therapy' OR (cognitive AND behavioral AND ('therapy'/exp OR therapy)) OR 'behavioral therapies, cognitive':ti,ab,kw OR 'behavioral therapy, cognitive':ti,ab,kw OR 'cognitive behavioral therapies':ti,ab,kw OR 'therapies, cognitive behavioral':ti,ab,kw OR 'therapy, cognitive behavioral':ti,ab,kw OR 'psychotherapy, cognitive':ti,ab,kw OR 'therapy, cognitive':ti,ab,kw OR 'cognitive therapies':ti,ab,kw OR 'therapies, cognitive':ti,ab,kw OR 'cognitive therapy':ti,ab,kw OR 'cognitive behaviour therapy':ti,ab,kw OR 'behaviour therapies, cognitive':ti,ab,kw OR 'behaviour therapy, cognitive':ti,ab,kw OR 'cognitive behaviour therapies':ti,ab,kw OR 'therapies, cognitive behaviour':ti,ab,kw OR 'therapy, cognitive behaviour':ti,ab,kw OR 'cognitive psychotherapy':ti,ab,kw OR 'cognitive psychotherapies':ti,ab,kw OR 'psychotherapies, cognitive':ti,ab,kw OR 'cognition therapy':ti,ab,kw OR 'cognition therapies':ti,ab,kw OR 'therapies, cognition':ti,ab,kw OR 'therapy, cognitive behavior':ti,ab,kw OR 'behavior therapies, cognitive':ti,ab,kw OR 'cognitive behavior therapies':ti,ab,kw OR 'therapies, cognitive behavior':ti,ab,kw OR 'therapy, cognition':ti,ab,kw OR 'behavior therapy, cognitive':ti,ab,kw OR 'cognitive behavior therapy':ti,ab,kw

#1 AND #2

CNKI 58

(pajinsen+pajinsenbing+pajinsenzonghezheng+pajinsenshibing+zhenchanmabi) AND (renzhixingweiliaofa+renzhiliaofa+xingweiliaofa+renzhixingweiliaofa)

CBM 102

(pajinsen OR pajinsenbing OR pajinsenzonghezheng OR pajinsenshibing OR zhenchanmabi) AND (renzhixingweiliaofa OR renzhiliaofa OR xingweiliaofa OR renzhixingweiliaofa)

WF 50

(pajinsen OR pajinsenbing OR pajinsenzonghezheng OR pajinsenshibing OR zhenchanmabi) AND (renzhixingweiliaofa OR renzhiliaofa OR xingweiliaofa OR renzhixingweiliaofa)

VIP 20

(pajinsen OR pajinsenbing OR pajinsenzonghezheng OR pajinsenshibing OR zhenchanmabi) AND (renzhixingweiliaofa OR renzhiliaofa OR xingweiliaofa OR renzhixingweiliaofa)
